# Content analysis of the Global Alliance for Nursing and Midwifery discussion forum: an online community of practice^*^

**DOI:** 10.1590/1518-8345.4878.3431

**Published:** 2021-09-03

**Authors:** Hillary Chu, Ashley Gresh, Valentina Bolanos, Nancy Reynolds

**Affiliations:** 1 Johns Hopkins University, School of Nursing, Baltimore, MD, United States of America.

**Keywords:** Collaboration, Content Analysis, Continuing Education, Knowledge Exchange, Professional Development, Online Community of Practice, Colaboração, Análise de Conteúdo, Educação Continuada, Troca de Conhecimento, Desenvolvimento Profissional, Comunidade de Prática Online, Colaboración, Análisis de Contenido, Educación Continua, Intercambio de Conocimiento, Desarrollo Profesional, Comunidad de Práctica en Línea

## Abstract

**Objective:**

to examine the usage and content of the Global Alliance for Nursing and Midwifery (GANM) discussion forum in relation to nursing and midwifery education and practice.

**Method:**

a qualitative conventional content analysis was performed. Subject lines from 1689 discussion board threads were extracted and used as the unit of analysis. *A-priori* codes were developed based on topical relevance (e.g. maternal health) and typical discussion board usage (e.g. announcing educational opportunities). Emerging codes were further identified while coding the data (e.g. infectious diseases).

**Results:**

the GANM discussion forum was used most frequently for information exchange (43.8%), such as dissemination of new information on evidence-based practice, and to announce educational opportunities (24.8%). The most frequently discussed topics were nursing (14.2%; e.g. the role of nurses in primary care, nursing education, etc.) and maternal health (13.8%; e.g. postpartum care, maternal mortality, etc.). Infectious diseases were discussed in 9% of threads, 40% of which concerned the current coronavirus pandemic.

**Conclusion:**

findings reinforce the utility of the GANM as a platform for professional development and continuing education. As a platform for disseminating empirical research, the GANM can be leveraged to have an influence on real-world, evidence-based practice.

## Abstract (pt)

**Objetivo:**

examinar o uso e o conteúdo do fórum de discussão da Aliança Global para Enfermagem e Obstetrícia (*Global Alliance for Nursing and Midwifery*, GANM) em relação à educação e prática de enfermagem e obstetrícia.

**Método:**

análise de conteúdo convencional qualitativa. Foram extraídas e usadas como unidade de análise linhas de assunto de 1.689 tópicos do fórum de discussão. *A priori,* os códigos foram desenvolvidos com base na relevância do tópico (por exemplo, saúde materna) e no uso típico de um fórum de discussão (por exemplo, anúncio de oportunidades educacionais). Códigos emergentes foram identificados posteriormente durante a codificação dos dados (por exemplo, doenças infecciosas).

**Resultados:**

o fórum de discussão da GANM foi utilizado com maior frequência para troca de informações (43,8%), como divulgação de novas informações sobre a prática baseada em evidências e para anunciar oportunidades educacionais (24,8%). Os tópicos mais discutidos foram Enfermagem (14,2%; por exemplo, o papel do enfermeiro na atenção primária, educação em enfermagem, etc.) e saúde materna (13,8%; por exemplo, cuidados pósparto, mortalidade materna, etc.). Doenças infecciosas foram discutidas em 9% dos tópicos, sendo que 40% estavam relacionados à atual pandemia de coronavírus.

**Conclusão:**

os achados reforçam a utilidade da GANM como plataforma de desenvolvimento profissional e educação continuada. Como plataforma para disseminar a pesquisa empírica, a GANM pode ser aproveitada para ter uma influência na prática do mundo real baseada em evidências.

## Abstract (es)

**Objetivo:**

examinar el uso y contenido del foro de discusión de la Alianza Global para Enfermería y Obstetricia (*Global Alliance for Nursing and Midwifery*, GANM) en relación con la educación y práctica de Enfermería y Obstetricia.

**Método:**

se realizó un análisis de contenido convencional cualitativo. Se extrajeron líneas de 1689 hilos de conversación en foros de discusión y se utilizaron como unidad de análisis. *A priori*, los códigos se desarrollaron sobre la base de la relevancia del tema (por ejemplo, salud materna) y el uso típico de los foros de discusión (por ejemplo, anunciar oportunidades educativas). Los códigos emergentes se identificaron con mayor precisión al codificar los datos (por ejemplo, enfermedades infecciosas).

**Resultados:**

el foro de discusión de la GANM se utilizó con mayor frecuencia para el intercambio de información (43,8%), como la difusión de nueva información sobre la práctica basada en la evidencia, y para anunciar oportunidades educativas (24,8%). Los temas más discutidos fueron la Enfermería (14,2%; por ejemplo, el rol de los enfermeros en la atención primaria, educación en Enfermería, etc.) y la salud materna (13,8%; por ejemplo, atención postparto, mortalidad materna, etc.). Las enfermedades infecciosas se discutieron en 9% de los hilos de conversación, de los cuales, el 40% refirieron a la actual pandemia de coronavirus.

**Conclusión:**

los hallazgos refuerzan la utilidad de la GANM como plataforma para el desarrollo profesional y la educación continua. Como plataforma para difundir investigación empírica, la GANM se puede aprovechar para influir en la práctica basada en la evidencia del mundo real.

## Introduction

Given that 2020 is the year of the nurse and midwife, it is worth highlighting the importance of having a competent and educated workforce across different system levels and settings^(1)^. This entails providing the most up to date information pertaining to evidence-based practice and opportunities for continuous professional growth as means to build upon the competence of nurses and midwives globally^(1)^. Educational information exchange requires collaboration at the intra and interprofessional level to foster professional development and collaboration^(1)^.

These strategies for maximizing education in nursing and midwifery are essential for building competency and preparedness, especially in times of outbreaks such as the COVID-19 pandemic. According to Brand^(2)^, nurses are key players in a country’s response to a national or international crisis. This means that they are not only providing care at the individual or population level, they are also improving health systems^(2)^. Thus, a commitment to continuous education is necessary to maximize the response of nurses in the face of a pandemic. To achieve such goals, an educational approach for a new infectious disease must address clinical knowledge, buildup of competencies, and accessibility to high quality, evidence-based material^(2-3)^. Online communities of practice can serve as an essential tool to provide access to up to date resources to respond to healthcare needs for nurses and midwives globally^(4-5)^.

In 2006, the Johns Hopkins School of Nursing PAHO/ WHO Collaborating Center established the Global Alliance for Nursing and Midwifery (GANM), an online community of practice that provides an innovative forum for knowledge exchange. Currently the GANM has over 4,000 members from 163 countries comprised of practitioners, researchers, and others. The GANM’s mission is to provide a platform for knowledge exchange globally, using low band-width technology.

The GANM Knowledge Gateway supports a discussion board and an online library to provide a mechanism for knowledge dissemination globally. The discussion board serves as an online forum for sharing information and ideas, particularly related to nursing and midwifery. What makes GANM’s discussion forum unique is that it is moderated by graduate students at Johns Hopkins School of Nursing. Moderators perform supportive tasks such as providing help to members, as well as administrative tasks such as deleting spam^(6)^. Of relevance to the current study, all messages posted to the GANM discussion board must first be approved by a moderator. Nearly all messages were approved, with the exception of posts that were selling a product or contained inappropriate content.

The objective of this study was to examine the usage and content of the Global Alliance for Nursing and Midwifery (GANM) discussion forum in relation to nursing and midwifery education and practice.

## Method

A qualitative content analysis was performed. This method was selected because it is commonly used in nursing studies and is a practical method for analyzing communication messages^(7)^. A qualitative approach was used to explore content areas discussed on the GANM discussion board. The content analysis is an appropriate methodology because it provides a “systematic and objective means of describing and quantifying phenomena”^(8)^. Using this approach, *a priori* codes were developed by the research team covering a range of maternal and child health topics and typical discussion board posts as identified by GANM moderators. New codes were added as new themes emerged. These codes were then grouped into categories derived from the data. One research team member coded all of the data and regular meetings were held with the research team to establish intercoder reliability.

Discussion board posts from the GANM Knowledge Gateway were first extracted for analysis from its inception in 2006 to October 2019. However, in early 2020, with the onset of severe acute respiratory syndrome coronavirus 2 (SARS-CoV-2) outbreak, members began sharing new information about the virus and the disease it causes, COVID-19. At the time, scientific knowledge about the virus and disease was still emerging, and concern about the spread of misinformation was growing. Because we were interested in the utility of the GANM forum in sharing evidence-based information, a second round of data extraction was performed in early May 2020.

As of May 2020, the GANM consisted of 4,036 members in 162 countries. A survey of GANM members conducted in August and September 2019 indicated that most respondents were licensed or registered nurses (36%), midwives (33%), or licensed advanced practice nurses [15% Certified Nurse-Midwife (CNM), 6% Nurse Practitioner (NP), 5% Doctorate of Nursing Practice (DNP), 2% Clinical Nurse Specialist (CNS), and 1% nurse anesthetist]. Twelve percent were public health specialists, including Master’s in Public Health and PhD. Sixty-two percent of respondents identified English as their primary language, 29% identified Spanish, and 24% identified another language, with Portuguese, French, Nepali, Vietnamese and Arabic being the most common other languages spoken^(9)^.

All threads from the inception of the discussion board in August 2006 until May 2020 were included. A new thread (or topic) begins when a member shares information or asks a question by posting a message with a subject line on the discussion board. Other members participate in the discussion by responding to the original post. Often, there are multiple conversations about similar topics, but each conversation has its own thread. Additionally, the same topic may have more than one thread. For example, a member may wish to share information about an upcoming conference by posting a message on the discussion board. They may send reminders to the community about the conference in the form of a new message, which would start a new, separate thread.

The text of the discussion board was imported into an Excel file, where the subject lines from each thread were extracted and used as the unit of analysis. The text of the thread, names of GANM members who participated in the discussion, and dates on which messages were posted were omitted, so any personal identifying information was removed for the analysis. The subject lines only were then copied into a separate Excel file, which was then imported into the qualitative data analysis software F4 Analyze. F4 Analyze was used to facilitate data management and analysis. It was assumed that each subject line reflected the content of its corresponding thread, and was written in the same language.

Additionally, threads with 20 or more posts were reviewed to identify topics that generated the most participation. When GANM members participate in a conversation thread, each response is recorded as a post within the thread. This analysis was done because in the method described above, each thread is counted exactly once. By examining threads with greater than 20 posts we were able to identify specific topics of interest, creating a fuller picture of what GANM members choose to discuss and engage with on the discussion board.

*A priori* codes were developed based on maternal and child health content areas and typical discussion board usage. For example, because the GANM is an online community of practice for nurses and midwives, maternal health was often discussed. Typical discussion board usage consists of such activities as announcing upcoming events, including educational opportunities (i.e., conferences, online courses, webinars, etc.); posting job vacancies; and sharing information in the form of practice guidelines, press releases, and newsletters. Threads related to GANM activities (i.e., new blog posts, additions to the online community library, and GANM surveys) were written by a moderator and coded as “GANM.” It should be noted that GANM threads represent instances of information exchange categorized under their own code because they were generated by the moderators. Coding moderator-generated threads separately allowed for the differentiation between conversations initiated by moderators and discussion board usage driven by members at large. Messages were also cross-coded to identify the language in which they were posted.

Emerging codes were further identified while coding the data. For example, many posts discussed infectious diseases, including HIV, Ebola, and COVID-19 (Topics that comprised less than 3% of the total discussion threads were coded under “other health topics.”). Each subject line could have more than one code; for example, a subject line announcing a GANM blog post about nursing during COVID-19 written in Spanish was coded under “GANM,” “nursing,” “infectious diseases,” and “Spanish.”

Additionally, subject lines that discussed health in a specific country or geographical region were coded under the corresponding WHO region: African Region (AFRO), Region for the Americas (AMRO), Eastern Mediterranean Region (EMRO), European Region (EURO), South-East Asia Region (SEARO), and Western Pacific Region (WPRO).

One research team member coded all of the data and conferred with the team where there was ambiguity to establish intercoder reliability. For example, threads about engaging men and boys in family planning were not initially coded under “maternal health” because they address male participation. However, after conferral, the authors decided that because these threads centered around family planning, “maternal health” was an appropriate code.

The study was exempted from IRB review; discussion posts were de-identified and analyzed anonymously.

## Results

Between August 2006 and May 2020, the GANM discussion forum had 1689 unique discussion board threads with approximately 20 new posts each month. Results are presented based on usage (e.g. how members used the discussion forum) and content (e.g. what was discussed) in order to provide a comprehensive exploration of the discussion board.

### Usage

GANM members primarily use the discussion board to share opportunities for professional development, which was the purpose of 85.5% of all threads. Professional development was subdivided into five categories: information exchange, continuing education, GANM posts, job announcements, and other. As shown in Table 1, the most frequent use of the discussion board was for information exchange (43.8% of all threads), such as dissemination of new information on evidence-based practice and sharing press releases, policy briefs, and newsletters. The second most frequent use was for announcing opportunities for continuing education (24.8%), including conferences, online courses, and webinars, which accounted for nearly half (46.5%) of all educational opportunities. GANM threads, which represent instances of moderator-generated information exchange, comprised 12.3% of all posts. These threads primarily disseminated new ideas and evidence-based information in the forms of expert blog posts and article summaries of empirical research published in open access peer-reviewed journals, respectively.

**Table 1:** Usage of the GANM discussion board

Professional Development	N	%
Information exchange	740	43.8%
Continuing education	419	24.8%
GANM* (moderator-generated)	208	12.3%
Job announcements	77	4.5%
Other	58	3.4%

* GANM = Global Alliance for Nursing and Midwifery

### Content

In terms of content, the most frequently discussed topics were nursing (14.2%) and maternal health (13.8%), as shown in Table 2. Discussions about nursing included conversations about the role of nurses in primary health care, workplace safety for nurses, disaster nursing, and advancing nursing in global settings. Announcements about such campaigns as the Year of the Nurse and Midwife and Nursing Now, and the State of the World’s Nursing Report were also included. Maternal health topics included family planning, postpartum care, maternal mortality reports, and more recently, COVID-19 and pregnancy and breastfeeding.

**Table 2:** Discussion board content areas

Topic	N	%
Nursing	239	14.2%
Maternal health	233	13.8%
Other health topics	178	10.5%
Midwifery	169	10.0%
Infectious diseases	152	9.0%
Women's health	106	6.3%
Technology	78	4.6%
Child health	75	4.4%

Infectious diseases were discussed in 9% of threads. Guidance around disease outbreaks were frequently shared, including guidelines for the Ebola outbreaks that began in 2013 and 2018, in West Africa and the Democratic Republic of Congo, respectively; the Zika outbreak that began in South America in 2015; and the current COVID-19 pandemic that began in 2019 in China. COVID-19 was specified in 40% of all threads discussing infectious diseases.

Midwifery comprised 10% of all threads, and included midwifery education, midwifery competencies from the International Council of Midwives, and information about the Virtual International Day of the Midwife. Threads about midwifery also tended to receive many messages, indicating that there was interest among members in discussing this topic.

The threads that generated the most discussion among members (greater than 20 posts) were entitled, “Doulas” (53 messages) and “Looking for collaborators” (52 messages), followed by, “Traditional Midwives (Untrained)” (50 messages) and “Direct entry midwifery programme” (44 messages), as show in Table 3. A thread entitled, “2020 Year of the Nurse, celebrating nursing and midwifery!” generated the fifth most number of responses (43 total messages), many of which advocated for the profession of midwifery to be recognized separately from nursing. For example, one contributor to the thread wrote:*“Fantastic we are celebrating nursing and midwifery but...midwifery should not be under the umbrella name of nursing. Globally midwifery should be recognised as a separate profession”*. In contrast, another member shared a different perspective, writing: *“I think historically the two professions have so much commonalities, deeply intertwined and complements each other. My inclinations is that we should focus on celebrating and rejoicing 2020 as the year of the nurses and midwives together given their historical linkage in many parts of the world, especially in developing countries!”*.

**Table 3:** Discussion board threads that generated the most discussion (N represents the number of messages within the thread)

Discussion Board Thread Title	N
Doulas	53
Looking for collaborators	52
Traditional Midwives (Untrained)	50
Direct entry midwifery programme	44
2020 Year of the Nurse, celebrating nursing and midwifery!	43
Information	33
Partograph: Does it Improve MNH* Outcomes?	31
HELP TO MOTHERS: fundal pressure	29
Medical Aid Films New Films on Identifying a Sick Child in the Community	29
Simulation Education and Research Dialogue	29
Controversy Surrounding Bed-Sharing	28
Digest of Greetings for 2014	28
Worsening Situation for Nurses and Midwives - Numbers Dropping	28
Alternativas no farmacológicas para el alivio del dolor en el trabajo de parto	27
RE: Request for primary studies on respectful maternity care	27
What is your position on Workforce Migration?	27
Institutional Violence against women in childbirth globally	26
HOPE Nurses Conference | HIV in Women - Announcements	25
New York Times Article: American Way of Birth Costliest in the World	24
Human Resources for Health and Nursing	23
"Essential medicines out of reach for most people"	21
International Day of the Midwife ideas	21
Invitation to participate in 3rd Trimester Screening Ultrasound project to prevent maternal and neonatal mortality	21
The World needs Midwives with Straight Hair More than Ever	21
Birth Centers and home births	20
News: Royal College of Midwives ends 12-year campaign against caesarians, epidurals, inductions and use of instruments	20

* MNH = Maternal and Neonatal Health

One hundred and fifty-six threads (9.2%) discussed health in a specific country or region. The most frequently discussed region was the Americas (AMRO; 37.8%). The African region (AFRO) was also frequently discussed (28.8%), as was South-East Asia (SEARO; 10.9%).

A minority of threads were written in Spanish (13.5%). While a handful of subject lines were written in languages other than English or Spanish (notably, Portuguese and French), these comprised a negligible percentage of threads.

## Discussion

The findings of this qualitative content analysis reinforce the utility of the GANM as a platform for professional development. Information exchange was shown to be a primary function of the discussion forum. This is consistent with previous research findings that online discussion groups for health professionals are used more frequently for information sharing, communication, and networking^(5)^. With a global membership of over 4,000 practitioners, policymakers, researchers, and students the GANM forum is both an impactful and efficient vehicle for disseminating important information, including practice guidelines from the WHO and other international and professional organizations.

As a platform for disseminating empirical research, the GANM can be leveraged to have an influence on real-world, evidence-based practice. In particular, the GANM is used to share practice guidelines and professional competencies from international organizations, an important function of online communities of practice^(4)^. The ability to learn from other health professionals’ experiences is also a valuable purpose of online communities as networking, knowledge sharing, and participation and interaction are initiated among participants^(5,10-11)^.

Opportunities for continuing education were plentiful and, due to the value that the nursing profession places on lifelong learning, significant. In addition to sharing real-world continuing education activities (conferences, online courses, workshops, etc.), the GANM facilitates several aspects of lifelong learning, including knowledge as a dynamic process, collaborative learning, appropriate learning environment, and the act of seeking opportunities for learning^(12-13)^. The discussion forum offers a means to implement lifelong learning by serving as an interactive space for members to access evidence-based information, ask questions, and share experiences, creating a dynamic learning environment and opportunities for collaboration. By sharing article summaries of open access peer-reviewed empirical research hosted on the online community library, the GANM also offers opportunities for practitioners to actively seek out new knowledge.

With contributing members from over 60 countries, the GANM is an innovative platform for connecting practitioners worldwide for potential collaboration. This is most singularly exemplified by a post entitled, “Looking for collaborators”, which generated over 50 responses. The fact that a call for partnership garnered interest from members across the globe suggests that there is enthusiasm among members in using the GANM discussion forum for networking purposes. Indeed, previous research has shown that networking is a primary reason that health professionals use online discussion groups^(5)^. Additionally, the level of engagement within the debate surrounding the topic, “2020 Year of the Nurse, celebrating nursing and midwifery!” demonstrates that the GANM discussion board offers a platform for real dialogue.

This study sheds light on the role of online communities of practice in sharing evidence-based information about emerging health issues. The COVID-19 pandemic is a global crisis that has ushered in an era of information sharing spurred by a collective sense of urgency. During this time, the GANM discussion board has been used to share evidence-based, peer-reviewed research articles regarding COVID-19 and such topics as pregnancy, maternal and neonatal health, and the health of vulnerable populations. Webinars from such authoritative organizations as the Consortium of Universities for Global Health, the IBP Network, and Johns Hopkins University have also been announced. Of relevance to nursing and midwifery, guidance from the WHO has also been shared, including protocols for maintaining maternal, newborn, child and adolescent health and resources for addressing violence against women during social distancing. Such online training materials help nurses and midwives build competencies, particularly in treating patients with COVID-19.

Limitations of this study include the lack of detailed demographic information about the GANM’s membership and possible lack of representation from other health professions. For example, doulas and untrained midwives were popular topics of conversation, but it is unknown how many doulas and untrained midwives participated in these discussion threads. Furthermore, the demographic information known about members (for example, their country) is self-reported. A limitation with the research design is that only discussion board thread subject lines were used for analysis without delving into the content of each post. Future studies should examine the content of each post to further explore the information delivered to the members of the online community of practice.

Understanding how this online community of practice is being used for knowledge dissemination is important to impact the advancement of scientific knowledge for nursing and midwifery. The GANM team can use this information to continue to provide a platform for knowledge exchange and to disseminate information that we now know is most discussed and utilized among members. In addition, this study provides insight for researchers and policymakers to leverage online communities of practice to ensure that the latest evidence-based research and practice is being disseminated to wide audiences.

## Conclusion

This conventional qualitative content analysis sought to understand how members of the GANM online community of practice use the GANM discussion forum for communication and knowledge dissemination. The findings of this study show that the utility of the GANM centers on opportunities for professional development, continuing education, and worldwide collaboration. Exchange of empirical research and high-quality, evidence-based information is also a key function of the discussion forum, as it helps practitioners build professional competency and combats the spread of misinformation.

In light of the present COVID-19 pandemic, the ability to access reliable clinical knowledge is especially critical for nurses and midwives to maximize their impact of care. Because of social distancing, it is even more critical to be able to access practice guidelines, training webinars, and evidence-based research articles from reputable sources online. Doing so allows nurses and midwives to build their competencies from the safety of their own homes while also slowing the spread of SARS-CoV-2.

The GANM discussion forum represents a transformative approach for knowledge acquisition during a global public health emergency with implications for nursing and midwifery practice. As a platform for disseminating empirical research, this low band-width technology offers nurses and midwives opportunities for professional growth that can have an impact on their real-world practice when caring for patients with COVID-19.
